# Non-invasive respiratory support in paediatric critical care: protocol for a cohort study emulating the FIRST-line support for Assistance in Breathing in Children (FIRST-ABC) step-up randomised clinical trial using routinely collected data from the Paediatric Intensive Care Audit Network (PICANet) database

**DOI:** 10.1136/bmjopen-2025-105500

**Published:** 2025-10-27

**Authors:** Elisa Giallongo, Orlagh Carroll, Padmanabhan Ramnarayan, Rebecca Mitting, Sarah E Seaton, Dermot Shortt, Alexina J Mason, David A Harrison, Richard Grieve

**Affiliations:** 1 ICNARC, London, UK; 2 Department of Infectious Disease Epidemiology and International Health, LSHTM, London, UK; 3 Department of Surgery and Cancer, Imperial College London, London, UK; 4 Paediatric Intensive Care Unit, Imperial College Healthcare NHS Trust, London, UK; 5 Department of Population Health Sciences, University of Leicester, Leicester, UK; 6 Department of Health Services Research and Policy, LSHTM, London, UK

**Keywords:** STATISTICS & RESEARCH METHODS, Paediatric intensive & critical care, Observational Study

## Abstract

**Abstract:**

**Introduction:**

The development of the target trial emulation (TTE) methodology has enhanced the conduct of non-randomised studies. By leveraging readily available routinely collected data, TTEs offer opportunities for complementing randomised controlled trials (RCTs), providing more precise estimates and improving the external validity of RCTs. To explore this potential, we selected a successfully completed RCT as a case study. In the FIRST-line support for Assistance in Breathing in Children (FIRST-ABC) step-up RCT, high flow nasal cannula (HFNC) was found to be non-inferior to continuous positive airway pressure (CPAP) in terms of time to liberation from respiratory support in the paediatric critical care setting. We will emulate the FIRST-ABC step-up trial using routinely collected data from the Paediatric Intensive Care Audit Network (PICANet) database.

**Methods and analysis:**

This is a protocol for a TTE that will use longitudinally collected data from the PICANet database. The study aims to emulate the FIRST-ABC step-up RCT using an observational study design in a frequentist framework. We will benchmark the results against the published trial. The study will apply a new-user design by selecting children admitted to paediatric intensive care units that started HFNC or non-invasive ventilatory support (as a surrogate for CPAP). The eligibility criteria and selected outcomes will reflect those of FIRST-ABC within the constraints of the available routinely collected data. We will use advanced quantitative doubly robust methods to minimise the impact of confounding by indication and allow for heterogeneity according to child characteristics. The analysis will be repeated using a Bayesian approach for follow-up research.

**Ethics and dissemination:**

The research received ethics approval from the London School of Hygiene & Tropical Medicine Research Ethics Committee. This study will expand the findings from the FIRST-ABC step-up RCT, providing additional insight from a large representative sample using real-world data. The frequentist and Bayesian approaches will enable a discussion about the advantages and drawbacks of the two strategies. The results will be disseminated to the research and clinical community and made accessible to the public. In addition, the study results will be used in future research, which aims to supplement RCTs with additional evidence from a TTE.

**STRENGTHS AND LIMITATIONS OF THIS STUDY**

- This study will emulate the FIRST-line support for Assistance in Breathing in Children step-up randomised controlled trial (RCT), applying advanced design strategies and doubly robust methods to mitigate potential bias typically associated with observational studies, both at the design and at the analysis level.
- By incorporating both frequentist and Bayesian approaches, the study will facilitate a comparative discussion of their respective advantages and limitations.
- Replicating a successfully completed RCT allows for a robust benchmark to evaluate the concordance between the target trial results and those derived from its emulation using routinely collected data.
- The study will serve as a case study within the broader context of integrating external evidence from target trial emulations into RCTs, with the potential to reduce sample size, increase precision and improve external generalisability.
- Eligibility criteria, study treatments and endpoints replication are constrained by the limits imposed by routinely collected data.

## Introduction

### Background

 Nearly 30% of children admitted to paediatric intensive care units (PICUs) have a respiratory diagnosis.^1^ Non-invasive respiratory support (NRS) is a form of treatment which incorporates various non-invasive techniques aimed at improving alveolar ventilation and oxygenation.^2^ The use of NRS in PICU has increased over time, in recognition of the complications associated with endotracheal intubation.^3^ Continuous positive airway pressure (CPAP) is a standard treatment in NRS, but in the past decade high flow nasal cannula (HFNC) therapy has been introduced as an alternative method, which is better tolerated by the patients and easier to handle by the staff.^4^

FIRST-line support for Assistance in Breathing in Children (FIRST-ABC) step-up was a pragmatic randomised controlled trial (RCT) conducted in the UK among 600 acutely ill children aged 0–15 years, who were clinically assessed to require NRS. The RCT found that HFNC was non-inferior to CPAP in time to liberation from respiratory support.^5^

RCTs can provide unbiased estimates of the relative effectiveness and safety of alternative interventions, but practical constraints preclude well-powered head-to-head RCTs of all alternative interventions for all relevant patient groups.^6 7^ A sample size calculated solely on the primary outcome will prove inadequate for important secondary endpoints and subgroup analyses, with potential serious implications such as erroneous identification of differential subgroup effects, which may lead to inappropriate provision or withholding of treatment.^8^ From the patient perspective, it is important to provide relevant answers for secondary endpoints and underrepresented groups, as noted by the patient representatives consulted during the design of FIRST-ABC. In addition, when RCTs encounter too many challenges to successfully be completed, they are terminated without adequately answering any of their research questions.^9^

In these settings, RCTs may benefit from being supplemented with additional data. Routinely collected healthcare data provides an alternative information source, offering opportunities for complementing RCTs, including increased strength for more precise estimation of secondary endpoints and subgroup analysis. However, inherent biases (eg, confounding by indication) and the quality and detail of data are a drawback to their use in this context. The recent development of the target trial methodology aims to mitigate some of the biases common to routine data by applying the study design principles of RCTs to non-randomised studies.^10 11^

This protocol defines a strategy to emulate the FIRST-ABC step-up RCT using routinely collected data. This RCT was deemed feasible to emulate using routine data from a high-quality healthcare database, the Paediatric Intensive Care Audit Network (PICANet).^12^ A successful emulation will be defined by comparable estimates with the target trial.^13^

This target trial emulation (TTE) will expand the findings from the FIRST-ABC step-up RCT, offering additional insights from real-world data, and will provide a case study for future work on assessing comparative effectiveness when combining evidence from an RCT and a TTE.^14 15^

### Aim

This study aims to emulate the FIRST-ABC step-up RCT using an observational study design, and benchmark the results against the published trial. We will use advanced quantitative methods to minimise the impact of confounding by indication and allow for heterogeneity according to child characteristics. The analysis will be repeated using a Bayesian approach for follow-up research.

### Objectives

The objectives of the present TTE using PICANet data are:

1. To describe baseline characteristics and treatment patterns for HFNC and non-invasive ventilatory support (NIV) as a surrogate for CPAP when used as the first-line NRS approach in critically ill children. CPAP is not recorded as a daily intervention in PICANet; however, NIV has been demonstrated to be an appropriate proxy measure for this study.
2. To evaluate the non-inferiority of HFNC, as compared with NIV, on the time to liberation from respiratory support, both overall (primary endpoint) and according to clinically important subgroups.
3. To estimate the effectiveness of HFNC or NIV on mortality at PICU discharge, rate of intubation at 48 hours and duration of PICU stay (secondary endpoints).

## Methods and analysis

### Study design

This is a retrospective cohort study using longitudinal routinely collected data from PICANet.^12^ The study emulates the FIRST-ABC step-up RCT.^16^ The study is planned to start in May 2025 and end in September 2025.

### FIRST-ABC step-up

FIRST-ABC was a master protocol of two pragmatic randomised trials to evaluate the non-inferiority of HFNC versus CPAP for NRS in paediatric critical care.^16^ For the present study, we will focus on the step-up RCT which includes critically ill children requiring NRS for an acute illness. This was powered for the primary endpoint: a sample size of 536 children was required to achieve 90% power with a one-sided type I error rate of 2.5% to exclude the prespecified non-inferiority margin of an adjusted HR of 0.75, and anticipating 5% censoring due to death or transfer. To allow for withdrawal/refusal of deferred consent, the total sample size included 600 children.^17^ Of the 600 children randomised between August 2019 and November 2021, 573 were included in the primary analysis to measure time to liberation from respiratory support using a frequentist approach. This used a modified intention-to-treat (mITT) population, which only included participants who initiated treatment. HFNC met the non-inferiority margin criterion (HR 1.03, 95% CI 0.86 to 1.22). Similar results were seen in the per-protocol analysis (HR 1.03, 95% CI 0.86 to 1.23). Secondary outcomes included mortality at critical care unit discharge (OR 1.22, 95% CI 0.32 to 4.62) and intubation at 48 hours (OR 0.99, 95% CI 0.61 to 1.62). A summary of the components of the FIRST-ABC step-up emulation is reported in table 1.

**Table 1:** Specification of the FIRST-ABC step-up emulation

Protocol component	FIRST-ABC step-up RCT	Target trial emulation
Eligibility criteria	Critically ill children requiring NRS for an acute illness were eligible if meeting the following:	Children included in PICANet between 1 August 2018 and 31 March 2022 and starting HFNC or NIV (as a surrogate for CPAP) will be eligible if meeting the following:
	*Inclusion criteria*	*Inclusion criteria*
	1) Admitted/accepted for admission to PICU/HDU.	1) Admitted to PICU.
	2) Age >36 weeks corrected gestational age and <16 years.	2) Age >36 weeks corrected gestational age and <16 years.
	3) Assessed by the treating clinician to require non-invasive respiratory support for an acute illness.	NA
	*Exclusion criteria*	*Exclusion criteria*
	1) Assessed by the treating clinician to require immediate intubation and invasive ventilation due to severe hypoxia, acidosis and/or respiratory distress, upper airway obstruction, inability to manage airway secretions or recurrent apnoeas.	1) Receiving invasive mechanical ventilation at treatment assignment.
	2) Tracheostomy in place.	2) Tracheostomy in place.
	3) Received HFNC/CPAP for >2 hours in the prior 24 hours.	NA
	4) On home non-invasive ventilation prior to PICU/HDU admission.	NA
	5) Presence of untreated air-leak (pneumothorax/pneumomediastinum).	NA
	6) Midfacial/craniofacial anomalies (unrepaired cleft palate, choanal atresia) or recent craniofacial surgery.	NA
	7) Agreed ‘not for intubation’ or other limitation of critical care treatment plan in place.	NA
	8) Previously recruited to the FIRST-ABC trial.	3) Previously recruited to the FIRST-ABC trial.
	9) Clinician decision to start other form of non-invasive respiratory support (ie, not HFNC or CPAP).	NA
	NA	4) Planned admission.
Treatment strategies	Children were randomised to HFNC or CPAP. Clinicians were permitted to switch from HFNC to CPAP (or vice versa) or escalate to other modes of non-invasive respiratory support or invasive mechanical ventilation if prespecified treatment failure criteria were met.	Children will be assigned to HFNC or NIV as a surrogate for CPAP.
Assignment procedure	Subjects were randomised 1:1 to treatment using a central telephone/web-based randomisation service available 24 hours/7 days per week. The randomisation sequence was computer generated and variable block sizes were used to strengthen allocation concealment. Randomisation was stratified by site and age (<12 months vs ≥12 months).	Participants will be assigned to either strategy they start at baseline. Randomisation will be emulated by adjusting for baseline covariates using advanced statistical methods.
Follow-up period	Starts at randomisation and ends at liberation from respiratory support.	Starts at initiation of HFNC or NIV and ends at liberation from respiratory support.
Outcome	*Primary endpoint*	*Primary endpoint*
	Time to liberation from respiratory support, defined as the start of a 48-hour period during which the child is free of all forms of respiratory support.	Time to liberation from respiratory support, defined as the start of a 2-day period during which the child is free of all forms of respiratory support.
	*Secondary endpoints*	*Secondary endpoints*
	Mortality at critical care (PICU/HDU) unit discharge.	Mortality at PICU discharge.
	Rate of intubation at 48 hours.	Rate of intubation at day 2.
	Duration of critical care (PICU/HDU) stay.	Duration of PICU stay.
	Duration of acute hospital stay.	NA
	Patient comfort.	NA
	Sedation use during NRS.	NA
	Parental stress at 24–48 hours.	NA
Causal contrast	Modified intention-to-treat and per-protocol.	Modified intention-to-treat.
Analysis plan	Analyses of the primary and secondary outcomes were performed according to randomisation group in all consented children who commenced any respiratory support, invasive or non-invasive, following randomisation (primary analysis set) and in all consented children who met the eligibility criteria and commenced the randomised treatment (per-protocol analysis set).	Analyses of the primary and secondary outcomes will be performed according to treatment group in all children who commenced HFNC or NIV, adjusting for all relevant baseline covariates using a doubly robust method.

CPAP, continuous positive airway pressure; FIRST-ABC, FIRST-line support for Assistance in Breathing in Children; HDU, High Dependency Unit; HFNC, high flow nasal cannula; NA, not available; NIV, non-invasive ventilatory support; NRS, non-invasive respiratory support; PICANet, Paediatric Intensive Care Audit Network; PICU, Paediatric Intensive Care Unit; RCT, randomised controlled trial.

### Data source

We will identify the study population using PICANet, an audit database recording details of the treatment of all critically ill children admitted to PICUs across the UK and Ireland.^12^ The PICANet database will be used to identify children eligible for inclusion, providing a representative population of admissions in PICU requiring NRS. The PICANet Admission dataset includes demographic and clinical data on all admissions, daily interventions and information on discharge.^18^

### Study population

The study population will emulate the FIRST-ABC step-up eligibility criteria within the constraints of the available routinely collected data and will include children admitted to PICU between 1 August 2018 and 31 March 2022, aged >36 weeks corrected gestational age and <16 years, and starting HFNC or NIV as a surrogate for CPAP within 2 days from PICU admission. Children will be excluded if any of the following criteria are recorded: intubation and/or invasive ventilation, tracheostomy, previously recruited in FIRST-ABC, planned admission.^16^

### Exposures

In FIRST-ABC step-up, children randomised to HFNC were started at a flow rate based on body weight. When a child was deemed ready for weaning, the flow rate was reduced by 50%. Children randomised to CPAP were started at a pressure of 7–8 cm H_2_O. When a child was deemed ready for weaning, the pressure was reduced to 5 cm H_2_O. Both HFNC and CPAP were delivered through devices and interfaces already used as part of routine care at sites. For both groups, the fraction of inspired oxygen (FiO_2_) was titrated to maintain peripheral oxygen saturation (SpO_2_) of 92% or higher. Clinicians were permitted to switch from HFNC to CPAP (or vice versa) or escalate to other modes of NRS or invasive mechanical ventilation if prespecified treatment failure criteria were met (FiO_2_ ≥0.60, severe respiratory distress, patient discomfort).

In this study, exposure groups will include children who started HFNC or NIV, as per daily interventions in the PICANet Admission Data Collection Form.^12^ CPAP is not recorded in the PICANet daily procedures and, therefore, it cannot be identified. As NIV cannot be used to record HFNC, we will use NIV as a surrogate for CPAP, providing treatment strategies for the TTE.^18^

The assumption that NIV could approximate CPAP was tested on the FIRST-ABC step-up population linked to the relevant PICANet sample using a previously reported strategy.^19^ Of the children with a recorded activity date that matched the randomisation date, or the following date, 86% of children with recorded NIV were assigned to CPAP in FIRST-ABC step-up and 86% of children with recorded HFNC were assigned to the same treatment in FIRST-ABC step-up.

### Covariates

We will use demographic and admission information to create a cohort of children that reflects the FIRST-ABC step-up sample.

Potential covariates collected at PICU admission that will be explored using directed acyclic graphs (DAGs) include, but are not limited to: age, site, ethnic group, sex, type of admission to unit, previous PICU admission, source of admission, care area admitted from, elective admission, main reason for admission, cardiac arrest before PICU admission, cardiomyopathy or myocarditis, severe combined immune deficiency, hypoplastic left heart syndrome, leukaemia or lymphoma, liver failure, acute necrotising enterocolitis, spontaneous cerebral haemorrhage, neurodegenerative disorder, HIV, bone marrow transplant receiver, systolic blood pressure, SpO_2_, FiO_2_, arterial PaO_2_, base excess, lactate, mechanical ventilation, CPAP, pupil reaction.^20^

In addition, other daily interventions as defined by the PICANet Manual will be assessed as potential covariates from PICU admission up to the start of treatment.^18^

### Outcomes

The following outcomes will be measured:

Primary endpoint:

- Time to liberation from respiratory support, defined as the start of a 2-day period free of all forms of respiratory support. Children discharged from PICU will be assumed to be liberated from all forms of support.

Secondary endpoints:

- Mortality at PICU discharge.
- Rate of intubation at day 2.
- Duration of PICU stay.

### Subgroups

Subgroup analyses will be performed to test for interactions between the effect of starting treatment and the following baseline covariates, as pre-specified in the FIRST-ABC protocol:

- Age (<12 months vs ≥12 months).
- SpO_2_:FiO_2_ ratio at baseline (within five categories at quintiles of the continuous variable).
- Reason for admission (bronchiolitis vs other respiratory vs cardiac vs other reason).

### Sample size considerations

In the design of the FIRST-ABC step-up RCT, it was anticipated that 508 observed events would achieve 90% power with a one-sided type I error rate of 2.5% to exclude the prespecified non-inferiority margin of an HR of 0.75. To account for censoring from death or transfer and refusal or withdrawal of consent, the target sample size was set at 600 children.^5^

The design of the TTE recognises that between 2019 and 2022 there were 14 875 respiratory admissions in PICANet.^21^ From a previous PICANet sample of 2 years, it was anticipated that more than 3000 children (of which 2/3 assigned to HFNC) would meet the pre-defined eligibility criteria and start the study treatments in the first 2 days of critical care admission. The primary outcome will be tested for non-inferiority using the RCT margin (corresponding to approximately a 16-hour increase in median time to liberation), and the secondary outcomes for superiority. Among the secondary outcomes, the rate of intubation at 48 hours was considered of high importance by patient representatives. With a proportion of 15.4% from FIRST-ABC step-up results, we anticipate that, with unequal group sizes, a two-sided type I error rate of 5%, and 90% power, the study will be able to detect a 4% absolute difference in proportions.^22^

### Statistical analysis (1)—frequentist approach

#### Planned analyses

The FIRST-ABC step-up primary outcome analysis was based on a mITT population, excluding children with no recorded respiratory support post-randomisation.^16^ To emulate the target trial primary analysis, the study will take a mITT approach, where each child will contribute to the original treatment group they started, irrespective of which treatments they may initiate later. Children will be followed until the date of their last recorded respiratory support or date of death.

To describe child characteristics, means, SD, medians and 25th and 75th percentiles will be calculated for continuous variables. Counts and percentages will be reported for categorical variables. The balance of baseline characteristics between treatment arms will be assessed.^23^

For the primary endpoint, the Kaplan-Meier method will be used for a crude comparison of time to liberation from respiratory support. To reduce the risk of bias due to model misspecification, doubly robust (DR) methods will be implemented for the analysis of the primary and secondary outcomes.^24 25^ This process involves fitting two models, one for the treatment and one for the outcome. The double-robustness property ensures statistical consistency of the estimator provided that at least one of the models is correctly specified.^26^

The DR methods that will be explored include augmented inverse probability treatment weighting (AIPTW) and targeted maximum likelihood estimation (TMLE).^27 28^ AIPTW improves the IPTW estimator by making use of the prognostic information in the conditioning set of the outcome model.^29^ TMLE is a substitution estimator that incorporates an outcome model and a propensity score model through a targeting step and implements non-parametric machine learning algorithms, which are more flexible than restrictive parametric models.^30 31^

Candidate methods will be investigated in terms of performance and ease of implementation, and details of the planned analysis will be included in the statistical analysis plan.

Adherence to the exposure will be measured as the proportion of children who switched to the other exposure, by treatment group. The percentage of children experiencing one or more protocol deviations will be compared between treatment groups using a χ^2^ test.^32^

#### Sensitivity analyses

To assess the risk of immortal time bias, a sensitivity analysis will exclude children who were assigned to the treatment on the day after their PICU admission. This will further align the timing of eligibility assessment with treatment assignment.

Additional sensitivity analyses will compare the primary method with other suitable strategies, including DR and non-DR methods, like G-computation.^33 34^ Where required, the balance of baseline analysis covariates between treatment arms will be assessed before and after propensity score weighting.^23^

To test the no unmeasured confounding assumption, a placebo test, also known as negative control, will be implemented.^35^,^38^ Single cubicle occupancy in the first 2 days of admission will serve as the negative control outcome.

### Statistical analysis (2)—Bayesian approach

#### Planned analyses

The primary and secondary outcomes will be re-analysed using a Bayesian approach. This will provide a comparison with the frequentist approach, and we will discuss the advantages and drawbacks of the two strategies. Bayesian alternatives to the planned statistical analysis (1) methods will be explored by performance and ease of implementation.^39^,^41^

For key outcome measures, the posterior distribution will be presented and summarised.^42^ Sensitivity of the results to different priors will be explored.^43^

The results from the Bayesian analysis will additionally serve for future work as outlined in the Future work section.

### Missing data

The amount of missing data is expected to be minimal. The level, pattern and reasons for data missingness will be explored, and the plausibility of the missing at random assumption for missing data will be considered (using clinical knowledge and DAGs).^44^ If required, in the statistical (1) approach, missing baseline variables will be imputed using the multivariate imputation by chained equations algorithm, with at least five imputations.^45^ In the statistical (2) approach, an appropriate Bayesian multiple imputation strategy for handling missing data will be used if required.^46^

### Validation of results against FIRST-ABC step-up

Results will be compared with the FIRST-ABC step-up findings in the mITT population.

To quantify the difference in the effect size, the standardised difference between the RCT and the emulation results will be calculated.

### Patient and public involvement

Patient and Public Involvement and Engagement (PPIE) was incorporated throughout this project. A patient/public/parent group has been formed, with consideration given to the National Institute for Health and Care Research (NIHR) INCLUDE framework, ensuring that under-served groups were represented.^47^ Focus groups were/will be held throughout the project including during results dissemination. Participants were/will be reimbursed according to NIHR guidelines.^48^

## Strengths and limitations

This TTE will benefit from the large available sample size provided by the PICANet database, as well as its representative coverage of the FIRST-ABC step-up target population.

FIRST-ABC step-up will be emulated within the constraints of routinely collected data. As in any observational study, confounding and selection bias are major concerns. We will mitigate these risks through the TTE design and a validated and rich covariate data set, which will enable the key trial eligibility criteria to be applied, and most potential confounders to be included in the adjustment.

The inclusion of healthier children compared with FIRST-ABC step-up could dilute the effect towards the null, while failing to exclude children who initiated treatment before PICU admission could artificially shorten the follow-up period, further biasing the effect towards the null. In both cases, the bias is assumed to be non-differential. Other non-replicable exclusion criteria are contraindications to the treatments, and their unavailability in PICANet is mitigated by the treatment initiator design.

Study treatments and endpoints replication is constrained by the limits imposed by routinely collected data, but preliminary checks have proved NIV to be a valid alternative to CPAP.

Because the primary outcome cannot be assessed beyond PICU discharge, we will assume that respiratory support ends at the time of PICU discharge. This assumption may introduce differential bias as HFNC is more likely than CPAP to be continued on the ward.^49^

The use of daily rather than hourly data may limit our ability to target a population fully comparable to that in FIRST-ABC and may hinder accurate identification of the treatment received. Strategies will be implemented to minimise potential differences between treatment groups.

Additionally, DR analysis methods will be implemented, and sensitivity analyses will be performed to assess the robustness of the findings. Missing data are expected to be minimal.

This study will be reported using the Strengthening the Reporting of Observational Studies in Epidemiology guidelines.^50^

## Future work

This emulation will serve as a case study for a project that aims to supplement RCTs that would benefit from additional information with observational data. To do this, we will use Bayesian statistical methods to combine evidence from RCTs and TTEs from routinely collected data. The novel methodology will respond to the pressing need for new methods to leverage all available information, in order to advance healthcare decision-making quickly and more efficiently, and benefit all patients, including underrepresented groups.

## Ethics approval

This is a secondary data analysis project, which will use de-identified data from the PICANet database and from the FIRST-ABC step-up trial. PICANet has support and approval from the Health Research Authority Confidentiality Advisory Group to process confidential patient information without consent for research purposes under Regulation 5 of the Health Service (Control of Patient Information) Regulations 2002 (‘section 251 support’ of the NHS Act 2006) for data collected in England and Wales. The reference is 21/CAG/0098 and support is reviewed annually. In addition, the research database has ethics approval granted by the East Midlands—Derby Research Ethics Committee (ref. 18/EM/0267) and annual reports are provided to the committee.^51^ PICANet applies the National Data Opt-Out to the PICANet research database to ensure that no children are included in any research projects where an opt-out has been applied.^52^ The FIRST-ABC master protocol of trials received a favourable ethics opinion from the NHS East of England—Cambridge South Research Ethics Committee (reference number: 19/EE/0185) and approval from the Health Research Authority (Integrated Research Application System (IRAS) number: 260536).^16^

The study protocol was reviewed and received ethics approval from the London School of Hygiene & Tropical Medicine (LSHTM) Research Ethics Committee (reference number: 31369).

The interpretation and conclusions contained in this protocol are those of the authors alone.

## Dissemination

The study’s output will be submitted to peer-reviewed journals for publication, and presented at scientific conferences. Parents/guardians of FIRST-ABC step-up participants who consented to be contacted for future research will also be informed of the study results. Data visualisations of key results and lay summaries will be published on the Intensive Care National Audit & Research Centre (ICNARC) and LSHTM websites as a resource to be shared with key stakeholders and as accessible information for the general public.
