# Supplementary material for: Non-invasive respiratory support in paediatric critical care: protocol for a cohort study emulating the FIRST-line support for Assistance in Breathing in Children (FIRST-ABC) step-up randomised clinical trial using routinely collected data from the Paediatric Intensive Care Audit Network (PICANet) database
Source: BMJ Open. 2025 Oct 27;15(10):e105500. doi: 10.1136/bmjopen-2025-105500 (PMC12570939; doi:10.1136/bmjopen-2025-105500)
Supplement: Supplementary file 1 [file bmjopen-2025-105500.reviewer_comments.pdf]

PEER REVIEW HISTORY

BMJ Open publishes all reviews undertaken for accepted manuscripts. Reviewers are asked to complete a checklist review form and are provided with free text boxes to elaborate on their assessment. These free text comments are reproduced below.

ARTICLE DETAILS

Title (Provisional)

Non-invasive respiratory support in paediatric critical care: protocol for a cohort study emulating the FIRST-line support for Assistance in Breathing in Children (FIRST-ABC) step-up randomised clinical trial using routinely collected data from the Paediatric Intensive Care Audit Network (PICANet) database

Authors

Giallongo, Elisa; Orlagh, Carroll; Ramnarayan, Padmanabhan; Mitting, Rebecca; Seaton, Sarah E; BE, Dermot Shortt; Mason, Alexina; Harrison, David A.; Grieve, Richard

---

VERSION 1 - REVIEW

**Reviewer**	**1**
**Name**	**Gunnarsdottir , Johanna**
**Affiliation**	**University of Iceland**
**Date**	**21-Aug-2025**
**COI**	**none**

Thank you for the opportunity to review this protocol to a target trial emulation of the FIRST-ABC step-up RCT. The protocol is very well written, and a plan to use advanced statistical methods is described well. I find it admirable to submit this study protocol for publication, and a positive precedent as this is often not done for cohort studies. It was also positive that the authors present a validation of the two treatment options to be tested in the study, and the registration was reasonably accurate in the PICANet database.

The first comment is about the term non-invasive ventilatory support that appears suddenly when stating the aims, instead of CPAP that was introduced on the pages before. At first, I found this confusing, but a good explanation was later given in methods (2.5). Perhaps, a short clarification could be provided on page 6.

As for other cohort studies the main concern is how easy or difficult it will be to balance the groups adequately. The protocol included a detailed plan about the statistical methods for balancing groups, but I found that the plan about what covariates to include in the models could have been better developed. The authors list part of the available covariates to be

used in the analysis, but they do not mention if important covariates are unavailable (although they mention that they will use a negative control to assess unmeasured bias). Further, they do not present any information from previously published work or clinical guidelines that are in use, that inform on selective use of either treatment option.

As it happens, I am an obstetrician with limited insight into the basis of the choice of high-flow or CPAP in different clinical situations, but my neonatologist college has informed me that CPAP is most often used when babies are born preterm <32 weeks but high-flow is more often used for babies born at later gestational age. Also, CPAP would often be the treatment choice for severely ill children which a very preterm born would be if infected shortly after discharge from neonatology ward. Although the cohort is limited to 36 weeks corrected gestational age, I still wonder if gestational age at birth should be included in the treatment model to fully balance the groups.

It seems obvious that the main driver of the primary outcome (time to liberation of respiratory support) will be the severity of the illness that indicated the admission. The authors do not describe in what level of detail this covariate is registered into the database. Also, the reasons for admission will likely be very different in so wide age group, with the youngest primarily admitted for infections, often few months after an initial stay in neonatal care in those very preterm born. The authors have a plan for some subgroup analysis that seems reasonable regarding different subgroups of patients, but the stratification planned regarding age is to separate children under 12 months or younger from those 1 year or older, not considering if they were preterm born or not (as I understand the text).

I am sure some additional exploratory analysis is needed to finalize the analysis plan, but I do not know if it is a standard practice to do this after the publication of such cohort study protocol. Again, I want to congratulate the author, for submitting this protocol and I am hopeful that this project will produce useful results.

VERSION 1 - AUTHOR RESPONSE

Reviewer: 1
If you have selected 'Yes' above, please provide details of any competing interests.: NA

Response to Reviewer 1
Thank you for your kind support and constructive review. We agree that the use of non-invasive ventilation as a proxy measure for CPAP, without appropriate explanation, may be misleading. As you suggested, we included a brief statement in Objective 1 (page 6), which is further elaborated in section 2.5, as you rightly noted.
The considerations you raised regarding the covariates to be used in the model are highly pertinent. One of the strengths of this study is the richness of the PICANet database, which includes several relevant variables for analysis. There are multiple strategies to guide the selection of appropriate model covariates, and we intend to follow clinical expertise, conceptual frameworks (e.g., directed acyclic graphs, as mentioned in the manuscript), and the covariates used in the target trial, FIRST-ABC step-up, most of which are available in the same or a similar format in PICANet. However, we believe it is necessary to explore all potentially useful covariates – and their availability – before finalising the

statistical analysis. For this reason, in the current protocol, we have listed the variables we intend to investigate, without emphasising any in particular. The final set of covariates, along with the strategies used for their selection, will be made publicly available alongside the study results.
Your point about including gestational age at delivery as a model covariate is also well taken. The reason this variable is not considered necessary is that children will be assigned the treatment on the same day they start it, which cannot occur before they reach 36 weeks of corrected gestational age. This criterion eliminates potential selection bias due to preterm delivery, ensuring that all included children have the same eligibility with respect to gestational age at delivery.
Changes to the manuscript
1. Acronyms and abbreviations defined in the title.
2. Abstract updated as per Instructions for Authors.
3. Strengths and limitations of this study 4th point re-worded.
4. Objective 1 (section 1.3) re-worded.
5. Planned start and end date included (section 2.1).
6. The negative control outcome has been replaced. This decision was made after careful discussion among the authors regarding the relationship between the previous negative control outcome (i.e., unplanned readmissions occurring 30 days or more from the index admission) and the treatment. The new negative control outcome, single cubicle occupancy in the first 2 days of admission, was deemed more appropriate.
7. Ethics and Dissemination have been moved to the main text (Sections 7 and 8).
